# Phenytoin-Induced Exanthematous Drug Eruption: A Rare Complication to Watch Out for!

**DOI:** 10.7759/cureus.58665

**Published:** 2024-04-20

**Authors:** Avinash Parepalli, Sourya Acharya, Sunil Kumar, Amol Andhale, Keyur Saboo

**Affiliations:** 1 Internal Medicine, Jawaharlal Nehru Medical College, Datta Meghe Institute of Higher Education & Research, Wardha, IND

**Keywords:** case report, complication, seizures, rash, phenytoin

## Abstract

*Phenytoin* is a first-generation anticonvulsant medicine that efficiently cures a wide range of seizures, including status epilepticus, complex partial seizures, and generalized tonic-clonic seizures (GCTS). The major advantage of phenytoin is that its neurological functions are preserved. Phenytoin works by inhibiting voltage-dependent membrane Na channels, which are essential to generate action potential. This function inhibits the positive feedback, leading to high-frequency repeated firing, reducing seizure spread in the focal region. A purple color rash on the chest, abdomen, and trunk developed in a 21-year-old female patient after being treated with phenytoin is being reported. The presentation, pathophysiology, and management are also reviewed.

## Introduction

Phenytoin sodium, an anticonvulsant, has been used to treat and prevent seizures since 1938. Phenytoin is a second line in the management of status epilepticus after intravenous (IV) benzodiazepines, as per the European Federation of Neurological Societies (EFNS) and the Epilepsy Foundation of America (EFA) [1].

Phenytoin inhibits voltage-dependent membrane Na channels [2], which are required to generate an action potential. This function reduces positive feedback, which causes high-frequency repetitive firing, limiting seizure spread in the focal area. However, phenytoin has been linked to major adverse drug reactions (ADR), such as dermatologic responses, severe hepatotoxicity, arrhythmias, and various hypersensitivity symptoms [3,4].

Extravasation (even soft tissue damage) from IV phenytoin treatment has been recorded since the 1950s. Comer et al. originally reported the link between IV phenytoin treatment and the quick development of discoloration, discomfort, and tissue necrosis in the distal leg via which the medication was delivered [5].

Increasing instances of phenytoin glove syndrome (PGS) have lately appeared [5], some including irreversible tissue injury or loss, raising concerns about the use of IV phenytoin. Phenytoin-induced rash is a documented adverse event that can develop in some people who take phenytoin. It is classified as a hypersensitivity reaction. It can take several forms, including Stevens-Johnson syndrome (SJS), maculopapular rash, and toxic epidermal necrolysis (TEN) [6,7]. Phenytoin-induced rash requires prompt medical treatment and should be reported to your doctor.

## Case presentation

A 21-year-old female presented to the casualty with the chief complaint of fever for five days, which is low grade and associated with fatigue and headache. She had two episodes of generalized tonic-clonic seizures, for which she was initially taken to the general hospital.

Upon arrival at the general hospital, she was semiconscious. Glasgow Coma Scale (GCS) was E2V2M5, blood sugar was 145 mg/dl), and she had an episode of generalized tonic-clonic seizures and post-ictal loss of consciousness. She was given a loading dose of phenytoin (Inj) 1000 mg over 20 minutes, and the patient was diagnosed as status epilepticus and was referred to a higher center for further management.

The patient was brought to our emergency room; on arrival, her GCS was E1V1M1, blood pressure (BP) was 110/70 mm hg, respiratory rate (RR) was 22/min, and heart rate was 110 bpm, which is hyperdynamic. Given her poor GCS and tachypnea, the patient was intubated in the emergency room. The patient had no past history of seizure disorder, hypertension, diabetes mellitus, thyroid disorder, or tuberculosis.

A computerized tomogram (CT) of the brain and magnetic resonance imaging (MRI) of the brain were obtained, and she was admitted to the medical intensive care unit. MRI and CT brain scans did not reveal any abnormalities. She was continued on IV phenytoin 100 mg thrice daily.

Cerebrospinal fluid analysis revealed lymphocytic pleocytosis. The total leucocytic count (TLC) was 40 cells/cumm, with a predominance of lymphocytes of 80%. Cerebrospinal fluid culture sensitivity was performed, suggesting no organism growth after 48 hours of inoculation. The diagnosis of viral encephalitis was made, and the patient was started on injectable antiviral therapy (acyclovir 500 mg IV thrice daily) along with other supportive care. Her lab parameters are shown (Table 1).

**Table 1:** The lab parameters of the patient TSH: Thyroid stimulating hormone; FT3: Free tri-iodothyronine; FT4: free thyroxine

Lab Parameters	Observed Value	Normal Range
Hb (Haemoglobin)	9.1 gm%	13-17 gm%
MCV (Mean Corpuscular Volume)	87.4 fL	83-101 fL
TLC (Total Leucocytic Count)	17800 cells/cu mm	4000 – 10000 cells/cu mm
Neutrophils	45%	40-60%
Lymphocytes	45%	20-40%
Monocytes	7%	2-8%
Basophils	1%	0.5-1%
eosinophils	2%	1-4%
Platelets	3.03 lakhs/cu mm	1.5 – 4.1 lakhs/ cu mm
Urea	19 mg/dL	19-43mg/dL
Creatinine	0.6 mg/dL	0.66-1.25mg/dL
Sodium	141 mmol/L	137-145 mmol/L
Potassium	3.6 mmol/L	3.5 – 5.1mmol/L
Calcium	7.6 mg/dL	8.4-10.2 mg/dl
Magnesium	1.6 mg/dL	1.6-2.3mg/dL
Phosphorous	3.6 mg/dL	2.5-4.5mg/dL
Uric acid	9.2 mg/dL	3.5-8.5mg/dL
Alkaline Phosphatase	78 U/L	38-126 U/L
ALT (Alanine Transaminase)	32 U/L	< 50 U/L
AST (Aspartate Transaminase)	35 U/L	17-59U/L
Albumin	3.2 g/dL	3.5-5 g/dL
Total bilirubin	0.5 mg/dl	0.2-1.3mg/dl
Conjugated bilirubin	0.1 mg/dl	0.0-0.3mg/dl
Unconjugated bilirubin	0.4 mg/dl	0.0-1.1mg/dl
RBS (Random Blood Sugar)	134 g/dL	90-140 g/dL
Ionic calcium	4.6 mg/dL	4.5-5.6 mg/dL
TSH	3.12 μ IU/ML	0.465-4.68 μ IU/ML
FT3	3.01 pg/ml	2.77-5.27 pg/ml
FT4	1.76 ng/dl	0.78-2.19 ng/dl
High Sensitive C-reactive Protein	2.22 mg/L	1.0 to 3.0 mg/L
Vitamin B12	346 pg/ml	239-931 pg/ml

**Figure 1:**
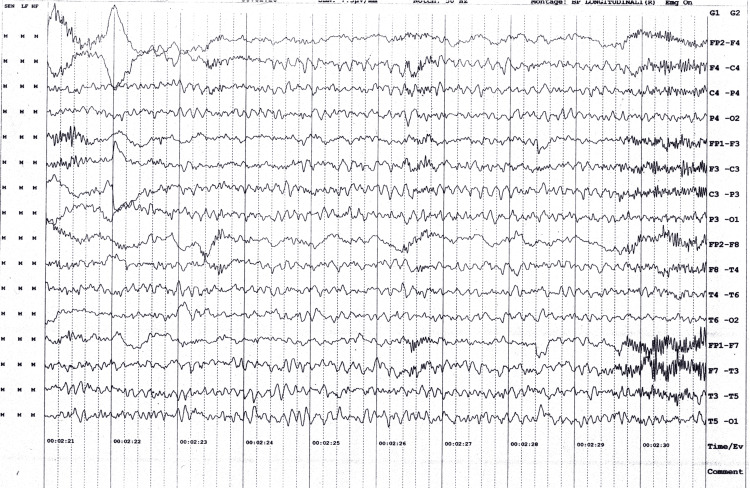
The electrical activity of the patient

An EEG was carried out, which suggested. The recording reveals rhythmic synchronous theta beta wave activity in both hemispheres at 7.5 microvolts, generalized slowing of theta beta rhythm, and intermittent sharp and high amplitude waves with burst suppression (Figure 1).

**Figure 2:**
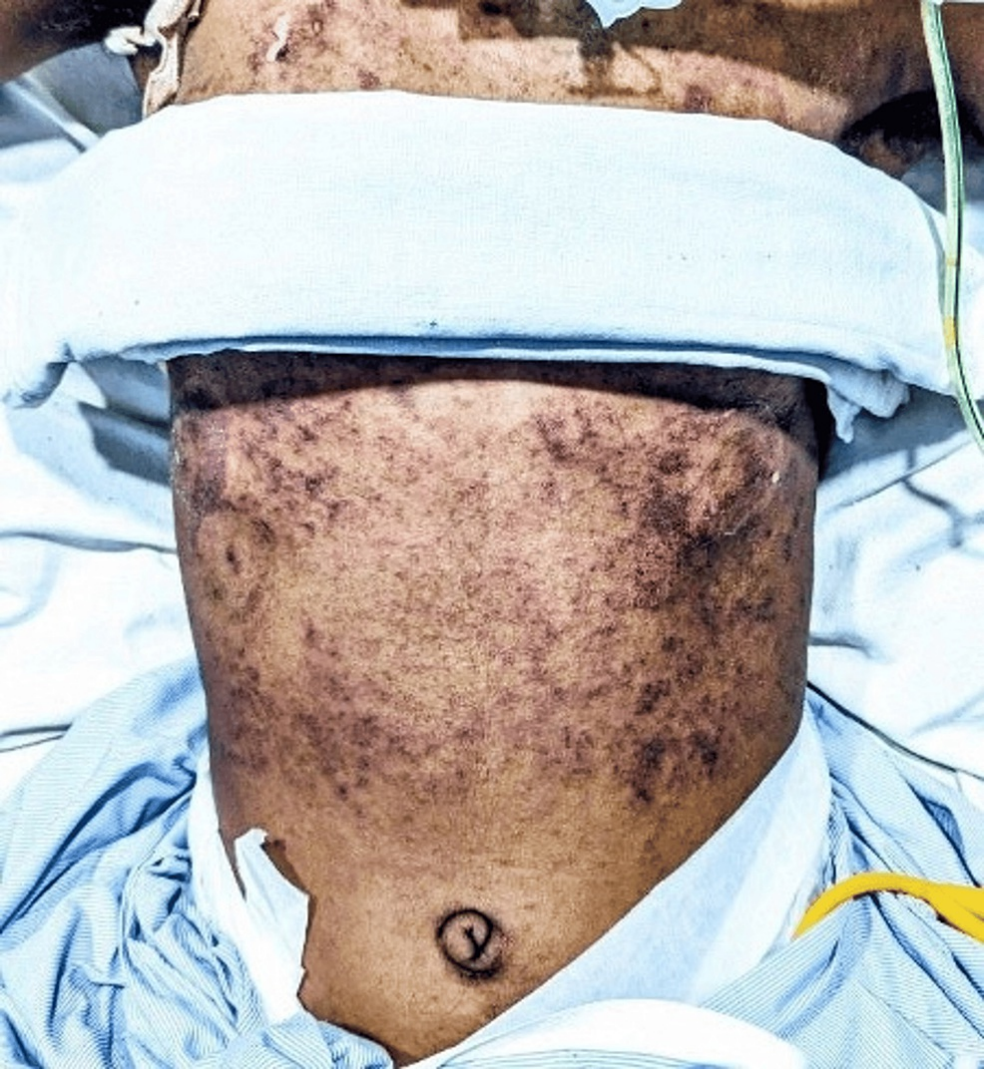
The patient's abdomen and chest demonstrate the appearance of a bluish-purple maculopapular violaceous eruption, which spread widely after four days of intravenous administration of phenytoin

**Figure 3:**
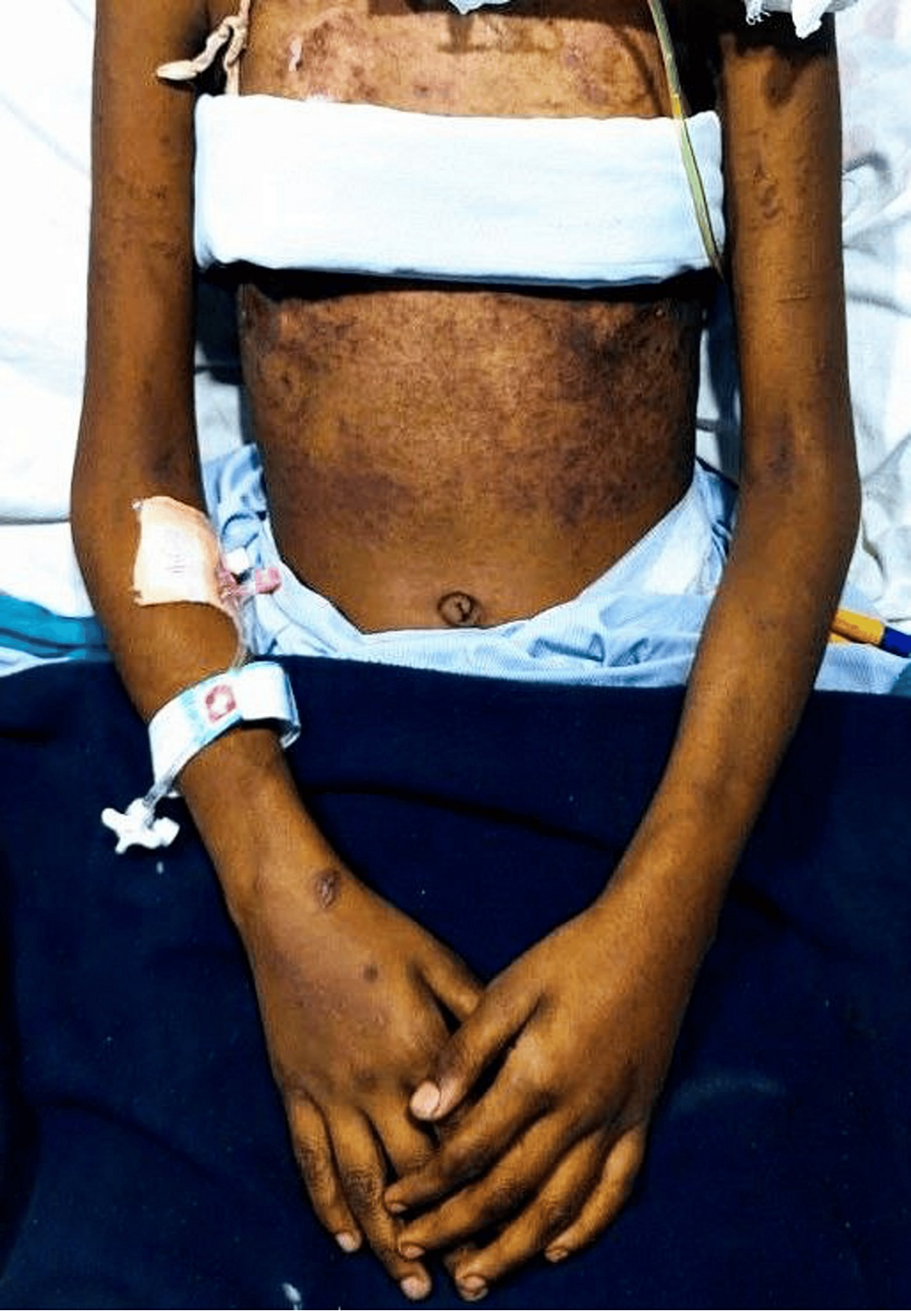
The widespread purple color maculopapular violaceous eruptions on the abdomen and chest, sparing the hands and peripheries

On day four of therapy, she developed a widespread itching maculopapular violaceous eruption on her lower abdomen. No crusts were evident, and mucosa was spared. On the next day, it evolved into bluish-purple scales with no evidence of bullae, blistering, or crust formation (Figure 2, 3). On examination, the patient's pulse rate was 98 beats/min, blood pressure was 110/70 mm Hg, and she was afebrile; no signs of pallor, icterus, cyanosis, clubbing, lymphadenopathy, and pedal edema.

Her lab parameters were repeated and suggestive of hemoglobin 8.4 gm/dl, total leucocytic count- 16600 cells/cumm, neutrophils- 49%, lymphocytes- 41%, monocytes- 6%, basophils- 1%, eosinophils- 3%.

**Figure 4:**
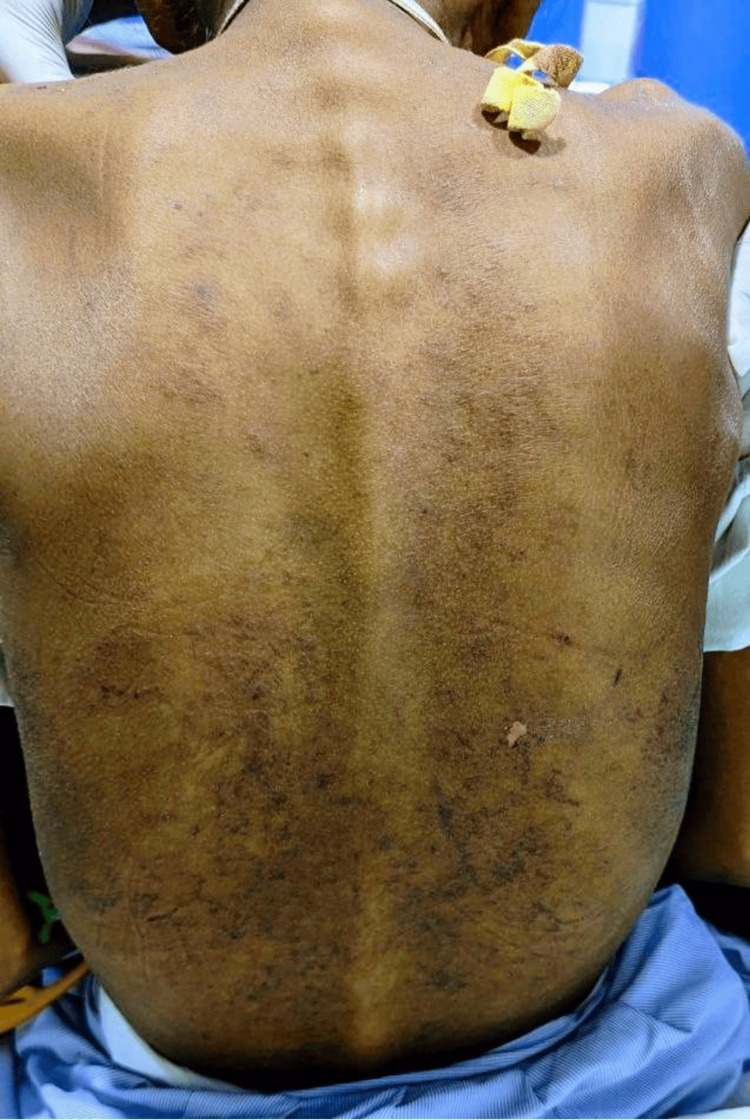
The trunk of the patient showing the widespread purple maculopapular violaceous eruptions that appeared after the intravenous phenytoin administration

The rash began to spread to the entire abdomen, and fresh scales appeared on the trunk (Figure 4). The patient was diagnosed with a rare complication due to an intravenous phenytoin injection, and she was stopped on the drug. Then, the patient was started on low-dose corticosteroids, such as dexamethasone (Inj) 4mg bd for five days, followed by 4 mg od for three days and local application of moisturizer. The rash began to decrease after seven days, and on follow-up after 1 month, there was no rash.

## Discussion

Phenytoin, an anti-epileptic medication, induces a hypersensitivity syndrome characterized by fever, rash, and lymphadenopathy. Other aromatic anti-epileptic medications, including carbamazepine, phenobarbital, and primidone, can also produce this problem. The incidence of anti-epileptic-induced hypersensitivity syndrome is 1 in 1000 exposures [8] and is linked to human herpesvirus-6 reactivation and seroconversion to human herpesvirus. The first indication is fever, malaise, and pharyngitis, sometimes with a strawberry tongue. An eruption occurs, displaying various forms of rash, erythroderma, or a generalized pustular eruption. Systemic involvement is prevalent, with the liver, kidneys, CNS, and lungs all possibly affected. Hypothyroidism may develop about two months after the initial symptom onset.

The most effective treatment for hypersensitivity syndrome is the rapid withdrawal of phenytoin. Phenytoin can be replaced with other anti-epileptics, such as valproic acid, but there is a risk of cross-reactivity and hepatitis [8,9]. Other anti-epileptics, such as lamotrigine and gabapentin, do not often cause phenytoin-induced hypersensitivity syndrome in patients. Hydration, antihistamines, H1-receptor blockers, and topical corticosteroids can all be used to help patients with phenytoin-induced hypersensitivity syndrome. Systemic corticosteroids should be administered for at least a month.

Hypersensitivity syndrome's pathophysiology is multifaceted. The conversion of flavonoids into hydrolyzed aromatic molecules (such as arenes oxides) results in hypersensitivity [10,11]. When detoxification is insufficient, hazardous metabolites may attach to biological molecules, resulting in cell death or an immunological response [12]. An enzyme deficit (epoxide hydrolase) causes hypersensitivity to flavonoids. Older black males are more likely to have this illness, which runs in families.

Phenytoin can cause cutaneous pigmentation, chloasma, melasma, acquired acromelanosis, global cutaneous depigmentation, porphyria, unmask acute intermittent porphyria, and unclassifiable porphyria [13-15]. Phenytoin seldom causes inflammatory skin illnesses; however, it has been associated with linear IgA bullous dermatosis (drug-induced) along with cases of sarcoidosis. It has also been observed that long-term phenytoin medication causes heel pad thickening. A maculopapular rash is seen in Rubeola, Rubella, Roseola, Exanthematous drug eruption, scarlet fever, etc.

## Conclusions

Phenytoin, an anti-epileptic drug, can trigger a hypersensitivity reaction with fever, rash, and lymphadenopathy. The most successful therapy is quick phenytoin withdrawal, with valproic acid being used with care in the case of hepatitis. Hydration, antihistamines, H-receptor blockers, and topical corticosteroids can all benefit individuals with phenytoin-induced hypersensitivity syndrome. The pathogenesis of hypersensitive syndrome is complicated, with phenytoin being transformed into hydrolyzed aromatic compounds, which cause cell death or an immune response. Older black guys are more vulnerable to this sickness, which runs in families.

Phenytoin-induced maculopapular violaceous eruption is an uncommon but significant adverse effect that can be caused by phenytoin. Maculopapular violaceous eruptions appear as little purple or red patches on the skin and may be followed by fever, itching, and blistering. It is also crucial to highlight that the phenytoin-induced purple maculopapular violaceous eruption is distinct from other rashes associated with phenytoin usage, such as drug-induced urticaria.
